# Understanding illnesses through a film festival: An observational study

**DOI:** 10.1016/j.heliyon.2019.e02196

**Published:** 2019-08-14

**Authors:** Carla Reigada, Salvador Martín-Utrilla, Pilar Pérez-Ros, Carlos Centeno, Anna Sandgren, Beatriz Gómez-Baceiredo

**Affiliations:** a University of Navarra, Institute for Culture and Society, ATLANTES Research Programme, Pamplona, Spain; b Institute of Health Research of Navarra (IdiSNA), Pamplona, Spain; c Catholic University of Valencia San Vicente Mártir, GRICPAL Research Group, Valencia, Spain; d Palliative Care Unit, Valencia Institute of Oncology, Valencia, Spain; e Faculty of Nursing, Catholic University of Valencia San Vicente Martir, Valencia, Spain; f Center for Collaborative Palliative Care, Department of Health and Caring Sciences, Linnaeus University, Växjö, Sweden; g School of Communication, University of Navarra, Pamplona, Spain; h Clínica Universidad de Navarra, Palliative Care Department, Spain

**Keywords:** Education, Sociology, Public awareness, Audio-visual, Observation, Healthcare, Illness

## Abstract

Audio-visual materials play a fundamental role in the context of education, care and clinical treatment, as they seem to have a high impact on public awareness. This study aims to describe what messages are perceived by the society at an International Festival of Short Films and Art on Diseases that may help to understand difficult topics, such as illness, dying and suffering.

Through an observational, descriptive, cross-sectional study, using full participant observation and an open, self-administered questionnaire, 32 short films were analysed during a healthcare art festival. Categories were developed using inductive content analysis. The message perceived by the participants, after the viewing of the shorts and reflection of the debates among the attendees, were considered in four categories: i) creative and positive education is possible; ii) awareness of preconception and practical duties; iii) meaning of life changes the experience of illness; iv) family and caregivers also experienced suffering. The short films are considered as an excellent tool to generate social dialogue and debate. Public events can be understood as an opportunity to acquire, in an emotional and critical manner, other competencies for public awareness. Together, they are capable of communicating difficult messages through a fast, positive, and creative way.

**What this study adds?**

• Festivals of art and other similar social events generate debates among people of different cultures, ages and status, encouraging the society to act actively and to adopt a critical discourse on difficult themes as death and dying, suffering and loneliness.
• The audiovisual art stimulates emotions which in turn generate empathic attitudes and behaviors.
• The understanding of complex subjects can be transmitted by art.

## 1 Introduction

The World Health Organization (WHO) warns of a society that lives surrounded by chronic diseases. The WHO report indicates that, while the death rate in Europe due to the diagnosis of diabetes, cancer, cardiovascular and chronic lung diseases has decreased, the rate of deaths due to suicide has increased (World Health Organization [WHO], 2017). These results reveal two critical problems; society is living longer with disabling diseases and, there is an increase in mental illnesses which are difficult to diagnose (Bartlett et al., 2017; Mulud and McCarthy, 2017; World Health Organization, 2017). More than ever the need emerges of providing adequate information and preparing society as to the implications of caring for people with illness (Sallnow et al., 2016).

Public awareness should ensure a proactive and positive tone and promote free reflection by society's members, on life, illness, suffering and death (WHO, 2018). Although awareness has increased, for example awareness of dying (Glaser and Strauss, 1965; Andrews and Nathaniel, 2015), public awareness is still low (Benini et al., 2011; McIlfatrick et al., 2013; Westerlund et al., 2018). A Swedish study about public awareness shows that 41% had no awareness of palliative care (PC) at all. Among the participants who had some knowledge (43%), they had received it via media and personal experiences (Westerlund et al., 2018).

Audio-visual materials (such films or documentaries) have been considered in scientific research as a potential educational tool for the transmission of messages to society (Carta, 2015; Das et al., 2017; Ramacciotti, 1960). Not being an objective art, cinema is often used as a didactic medium in pre- and postgraduate student training, both in the areas of social and health sciences (Dickens et al., 2018). One of the goals of audio-visual materials is to awaken feelings and the critical sense of the recipient by promoting collective reflection on a specific topic (Helgevold and Moen, 2015; Rieger et al., 2018). Movies dealing with death and dying are increasing and there seems to be a considerable audience who show interest in them (Drukarczyk et al., 2014).

Also, festivals and cultural/social events have been acquired a growing interest in social research. Symbolically, these multifaceted and creative spaces represent the cultural identity that can be created around a community, a sense of belonging to a particular interest (Mhlongo et al., 2018; Sowa et al., 2018). Tjora (2015) revealed that “festival space” has already gained recognition by observational studies in the areas of psychology and sociology as well as communication and education. In it, the experience of seeing, feeling, hearing and interacting, in a single space on a particular field, allows individuals to gain knowledge, solidifying values and relationships (Tjora, 2015). Film festivals allow people to attend an event with the aim of enjoying, interacting and having fun. Moreover, this type of event offers a creative, innovative, unexpected and open reflection space (Mackellar, 2013).

Audio-visual art can be a way of combatting the lack or misuse of information on difficult topics, and a social event (e.g. festival) can allow the spread of truthful information through various types of messages, generating social debates (Mhlongo et al., 2018). Together, they can facilitate the expression of feelings, experiences and thoughts that may help society to assume an active attitude facing, reflecting and discussing topics that they tend to avoid, as is the case with “death and dying” (Mhlongo et al., 2018).

## 2 Model

Three theoretical concepts have contributed to the design of our study. The ability model of Salovey and Mayer (1990) shows us that social and emotional learning provides necessary skills for self-knowledge and empathy, as well as promotes interpersonal relationships and facilitates decision making in conscience and in a constructive manner. The model of Steiner and Perry (1997) also defends that emotional education promotes basic capacities in the human being as the ability to understand emotions, the ability to express them in a productive way and the ability to listen to others and feel empathy. At last, the model of learning by observation, advocated by Bandura (1977), explains that emotions exert a great force on our thinking and our behavior. This theory defends that the observation has an instructor effect since emotions will activate cognitive abilities and a facilitating effect since the visualized and sensed stimuli will enhance behaviors.

Based on this, we agree with Lowman (1984, p. 101) that emphasizes that “students learn images as well as words, and images are more easily remembered especially if they are vivid and emotionally tinged.” Becoming aware of the importance of including emotional education in the process of lifelong learning, is the key to have an active society with multiple abilities, enhancing their social and emotional intelligence.

This study aims to describe the perceived messages by the society on short films at an International Festival of Short Films and Art on Diseases. It is possible that visual materials and social events can contribute to higher public awareness about illnesses?

## 3 Method

### 3.1 Design

Cross-sectional observational study design to measure the participant's perception of messages, at a given point in time, in a public festival (Emerson et al., 1995).

### 3.2 Context: the festival

The International Festival of Short Films and Art on Diseases (FICAE) is held annually in Spain, and it is unique in the world since it allows viewing of digital works of art for one week, is free of charge and is accessible to all groups of society (Festival Internacional de Cortometrajes y Arte sobre Enfermedades, 2018).

The International Festival of Short Films and Art on Diseases (FICAE) took place from 23^rd^ February to 3^rd^ March 2018 at the Institut Valencià d'Art Modern (IVAM), a museum and art center in the heart of the city of Valencia in Spain (Festival Internacional de Cortometrajes y Arte sobre Enfermedades, 2018). Valencia has about 822 000 inhabitants (World Population Review, 2018). Considering geographical, cultural and economic factors, this festival intended to create a reflexive space to clarify concepts and promote social debate on illness and its context.

The FICAE festival included a total of 62 short films from 26 different countries (16 from Europe, seven from America and three from Asia). These short films were distributed in 15 sessions, making up the 43 h of the festival. The duration of each session was 3 h with a median of five shorts per session followed by a discussion with the audience and keynote speakers.

Given the heading of each session, the research team decided to choose seven of the nine sessions of the official program of the festival. We considered the follow thematic sessions: 1) “Cancer”- one of the diseases that most concerns society; we all have a family member or friend who has fought this disease; 2) “Psyche 1” - there is a significant stigma about mental illness; this section focuses on disorders of the psyche, from the point of view of the patient or their environment; 3) “HIV AIDS” - one of the most socially stigmatized diseases, despite the great medical and social advances; 4) “Caregiving” - cantered on the figure of the caregiver as a fundamental part of the life of patients; 5) “Memory” - cantered on diseases that affect memory, especially Alzheimer's; 6) “Health Contexts” - selection of films about the health environment; and 6) “Parkinson” - audio-visual material on Parkinson's disease. We excluded Psyche2 and Miscellanea sessions, as they included topics presented in other sessions.

### 3.3 Data collection: the participants

Clinical and social professionals from the Valencia region were selected to be participants (full participant observation) considering their experience and familiarity with situations of chronic and advanced disease. The participants were invited by phone and email by one of the study's researchers. Information about the study, including informed consent, was sent to 19 persons. All agreed to participate in the study: five physicians, eight nurses, three social workers, one psychologist, one journalist and one physiotherapist; participants were aged between 22 to 47; the majority were female (n = 14), working as clinicians (n = 9) or in the academic setting as professors, researchers (n = 7), or students (n = 3). At least two participants from different professional profiles (health and social sciences) were selected for each session. After each session, a small group discussion was held among the participants to answer the research question on what type of messages about illness were being transmitted to society through the short films.

An observation form was used to register the observations. It was adapted from a questionnaire developed by the ATLANTES research group to characterize the nature of the content and specific issues on PC and end-of-life care in print media (Carrasco et al., 2007). It starts with a space for the free description of the scenes, dialogues, expressions, images, music, silences, attitudes or other significant aspects of each movie and another space to register the summary of discussions afterwards. The observation form also contains three direct-response questions on: a) the participants’ personal opinions regarding the general assessment of the short film (range 1–5), b) whether they would use the film as material to educate students (Yes/No) and c) if from their point of view, the session emitted a positive or negative message about severe illness.

### 3.4 Data analysis

Inductive qualitative content analysis was used to analyse the data (Mayring, 2000). Coding of the observation forms was conducted separately by CR and BG in an on-going process of constant comparison. Categories were developed and later discussed together in the research group (1^st^ round) considering the descriptions of film sessions and short film synopsis. After detailing the categories and sub-categories, with direct descriptions by the participants, the research group discussed (2^nd^ round) the final categories to reduce potential bias and increase internal validity. The ‘Consolidation Criteria for Reporting qualitative Studies’ (COREQ) guidelines were followed to ensure rigor in our research. We undertook a wide search of bibliography and analysis of relevant publications, and we selected specific criteria to lead study design, results, analysis and interpretations (Tong et al., 2007).

### 3.5 Ethics approval

Ethics committee approval was not required for this study.

## 4 Results

In eight sessions, 19 participants analysed 32 short films with a total of 18 h viewing time. In total, 56 completed observation forms were obtained from the participants. The Cancer, Psyche, Caregiving, Memory and Parkinson's sessions were those that recorded a higher number of independent observations per short film, facilitating discussion afterwards and the quality of the analysis (see Tables 1 and 2).

**Table 1:** Description of the included film sessions, short films and observations.

Thematic sessions and shorts films (codes)	Number of short films visualized	Number of observers attending the thematic session*	Total of evaluations forms collected*
**Cancer** Anna (CA1); Il Silenzio (CA2); In Fine (CA3); Lonely Hearts Club (CA4); Menos del 1% (CA5); Mom & Dad (CA6); Motion Pictures (CA7); Seré la semilla (CA8); **Annunciation (CA9)**	9	7	19
**Psyche** Cold Void (PS1); Grand Huit (PS2); Gris (PS3); Hum (PS4); Juin Juillet (PS5); Pipe Dream (PS6); Ulyst (PS7)	7	3	11
**HIV- AIDS** In the Wall (HI1); Plo Gladini (HI2); Postive Youtubers (HI3); The Condom Man (HI4); You Can't Hide From The Truth (HI5)	5	3	6
**Caregiving** Close Relations (C1); Сторге (C2); El camino al alba (C3); Me gustaría que esto fuera una pesadilla (C4)	4	4	7
**Memory** Head Above Water (M1); Julia y Manuel (M2); Memory (M3)	3	3	6
**Health Contexts** Desayuno con pastillas (H1); ¿Para qué Dios si tenemos padre? (H2)	2	3	3
**Parkinson** Salvado de las aguas (PA1); Feats of Modest Valour (PA2)	2	3	4
**Total**	32	26	56

*some observers observed more than one short film per session.

**Table 2:** Descriptions of film sessions, short films synopsis and codes used in data analysis.

THEMATIC SESSION/Short films	Film Synopsis (adpt. from Ficae Website)	Short film code (for data analysis)	Observer code (observers/short)
**CANCER**			
**Anna**	A conversation between two friends, where they deepen on the fantasies of their youth. For one of them, a patient with terminal cancer (nurse), the future is nothing but life after death. A simple and honest portrait that leads us to understand those people who have already assumed their end with dignified serenity.	CA1	P2; P6
**Il Silenzio (The Silence)**	Demonstrating the drama of cancer and refugees, a Kurdish girl is supposed to translate what the doctor says to her mother during her visit to the doctor, but she keeps silent. It is the staging of fear that affects many families, facing the terrible reality of cancer. However, it is evident that the “silence” does not resolve problem.	CA2	P2; P6
**In Fine**	Facing a certain death is something impossible to imagine until you find yourself in that situation. However, this documentary produced with the use of an elegant black and white (even when part is colour shot), shows the difficult task of expressing feelings and invites viewers to better understand terminal cancer patients. It is shocking how everyone changes their priorities at end-of-life.	CA3	P1; P4; P6
**Lonely Hearts Club**	Talks about cancer survivors, those who have overcome the disease and have to keep going with the consequences. The protagonist, a girl who attends a quick dating session for people who have overcome the disease, uses cynicism as a mechanism of protection towards others, but she will realize that this attitude does not help.	CA4	P3; P5; P6
**Menos del 1% (Less Than One Percent)**	No one is exempt from cancer. We always tend to think that it is not going to affect us. In tone of casual comedy, Flavio does a mammogram to support his wife morally. The result is as surprising as it is dramatic.	CA5	P4; P6; P7
**Mom & Dad**	The parents of Iranian director Shirin Qaredaqi died of lung cancer and she documented everything. What we achieve in our lives becomes our memory and may even prophesy the future. From a personal look and large doses of intimacy, Qaredaqi takes us on a journey into the past, showing the pain of seeing the closeness of the death of the people we love.	CA6	P6
**Motion Pictures**	“Explores the harsh reality of cancer and its effects physically and emotionally. Cancer is one obstacle that seems unsurmountable, but with a creative spirit, it is only fuel to create something bigger than yourself.” (http://animatedshortfilms.net	CA7	P6
**Seré la semilla (I Will Be The Seed)**	A poetic portrait of a dreamer. A construction technician, baker and actor, uses flour, plants and seed to introduce him. Luis faces the fight against the disease in his own way, with passion and optimism. Just as he would face a marathon, he breathes deeply and run.	CA8	P6
**Annunciation**	After a night of partying, Ceren confesses to her husband, that she has stopped taking the contraceptive pill because she wants to have a child, but Ali has a hidden secret from her past. An unsuspected confession that will lead Ceren to a crossroads of legal doubts, moral, fears, jealousy and finally, generosity that makes us question what we would do in his skin.	CA9	P3; P5
**PSYCHE**			
**Cold Void**	Niklas Kvaforth explains, in his own words, that he suffers from schizophrenia and bipolarity, which has led him to suffer severe depressions. At age of 4 he saw his mother commit suicide. An extreme life that is revealed in his multiple tattoos and scars. A first-person testimonial, which becomes a positive surprise when you get a glimpse of the person behind the disorders.	PS1	P6
**Grand Huit (Hide and Seek)**	It portrays psychiatric hospitals and their impact on the life of patients and their families, which brings us closer to bipolar disorder in a realistic way. An attempt to fight the public's fears and “clichés” about mental institutions and clinical places, usually unknown to the rest of society.	PS2	P6; P8
**Gris (Gray)**	A journey into the mind of a person with bipolar disorder in the depressive phase who has just attempted suicide. During a conversation with the psychiatrist, we are witnessing the most intimate feelings and thoughts of this person.	PS3	P6; P9
**Hum**	An artist suffering from schizoaffective disorder, he has been able to channel his obsessions towards musical creativity, becoming a genius of overflowing talent.	PS4	P6
**Juin Juillet (June July)**	Eva is a young patient, voluntarily hospitalized for two months due to her psychiatric issues. We witness the evolution of Eva's relationship with herself, her friends and even with the institution itself.	PS5	P6; P9
**Pipe Dream**	We enter the mind of a man in a psychiatric hospital, while his performing a musical show for his companions. The obvious interest of the director is to create a totally emotional experience, showing what happens in the patient's mind and what actually happens in the hospital's conference room.	PS6	P6; P8
**Ulyst** (**OverLove)**	A young woman who adores her little brother cannot avoid a series of invasive thoughts related to harming him. She is aware of that and decides to tell her mother, but she does not understand what is happening to her. Communication is the key to detect disorders and help patients to understand what happens to them and handle those situations.	PS7	P6
**HIV- AIDS**			
In the Wall	Ana is a heterosexual university student, who has erected an emotional wall to isolate herself from others and not be rejected after discovering that she was HIV positive, as a result of a sporadic unprotected relationship. It will be her friend Lucía who tries to make her see that we live no longer in the 80's, that with the information and adequate protection she can continue to be what it is, a girl with desire to live, to grow, to meet people, to have fun.	HI1	P9
**Plo Gladini**	The young Mitja returns to Slovenia with his boyfriend Bojan. There is something that he must tell him, but he cannot find a way to approach the subject. Communication and sincerity are the pillar of any relationship, but with special relevance when it comes to sexually transmitted diseases.	HI2	P10
**Postive Youtubers**	A group of Brazilian youtubers affected by HIV explain to their followers what their new life is like since they use retroviral treatment. It is presented as a need for the normalization of this disease.	HI3	P6
*The Condom Man*	A real case happened in Ireland during the 80s, when condoms could only be obtained under medical prescription. In that context, a group of people were infected by blood transfusions. A hostile heroic and anonymous struggle against the passivity of the government.	HI4	P9
**You Can't Hide From The Truth**	It Shows the issue on AIDS in Africa, where the reality is that the seropositive people do not recover their life thanks to medical advances. The film features a blind musician from Zimbabwe, survivor of a successful band of 32 musicians, of whom 29 died of AIDS.	HI5	P6; P10
**CAREGIVING**			
**Close Relations**	Having a dependent person at home can generate partner conflicts. The reality of a son, overwhelmed by the responsibility of taking care of his mother. His wife, who did not agree to have her mother-in-law at home (perhaps because of some quarrel from the past) will put the relationship to a limit of the rupture. One of the two will have to yield.	C1	P17
**Сторге (Storge)**	A daughter who must take care of her mother affected by Alzheimer's. Transmitting feelings of loneliness, the “storge” defines the natural love of a daughter based on commitment and affection. But the daughter cannot forget that there is a life out there, a life that she is losing.	C2	P12; P17
**El camino al alba (Into the Dawn)**	A dedicated Mexican nurse decided to consecrate her life to take care of terminally patients without resources and, often, without family. The sweet Juanita is the refuge of this people that spend the last days in peace. A sincere and emotional tribute to all those who with unlimited generosity, give their love to others.	C3	P16; P17
**Me gustaría que esto fuera una pesadilla (I would like this to be a nightmare)**	César and Angélica, two brothers with Morquio syndrome, who together with their sister Loretto, care delicately for their mother, affected by an advanced Alzheimer's disease.	C4	P17; P18
**MEMORY**			
**Head Above Water**	A husband caregiver of an Alzheimer's patient falls into the bathroom and it is his wife who must help him. The inability of her to carry out simple tasks such as bringing the phone to save him, will lead her husband to consider the need for professional help. This short, shows how many elderly spouses are overwhelmed when they must take care of each other.	M1	P15; P17
**Julia y Manuel**	Julia likes pigeons and the sun. Manuel likes listening to his favourite songs on the radio. Julia tries to seduce Manuel and Manuel finds Julia very attractive. They are not two strangers; they are a couple of many years. In this film, the uninhibited behaviour of people with senile dementia is shown with humour, but with great realism.	M2	P17; P19
**Memory**	Elder has left home to buy a toy for his grandson. When he wants to return, he cannot remember the address of his house. He is forced to take a walk through the city that will make him remember sensations of his childhood. This is one of the characteristics of Alzheimer's, the prevalence of long-term memory over short memory.	M3	P15; P19
**HEALTH CONTEXTS**			
**Desayuno con pastillas (Breakfast with Pills)**	A senior couple are having breakfast in the garden of their house. A bag full of pills in on the table. A story of a woman who must follow a routine of taking medication due several clinical issues, and a husband who helps her not to get lost.	H1	P7
**¿Para qué Dios si tenemos padre? (Why God if we have a father?)**	A family business building coffins. Nobody is in this job for vocation; however, it is interesting to discover what personal motivations have led each member to follow the family business. A different approach to the subject of death.	H2	P11; P17
**PARKINSON**			
**Salvado de las aguas ​ (Saved from the waters)**	A first-person portrait of an artist, athlete, sportsman, adventurer and doctor from Mexico, who has Parkinson's. Told through his artistic work and his memories, Moisés continues to create art with his hands despite the motor difficulties.	PA1	P11; P17
**Feats of Modest Valour**	Tom, Brian and Milena live under a medication regimen and the challenging physical reality of living with Parkinson. Meanwhile, a team of dedicated scientists in Galway, Ireland, is developing a new medical device that could halt or even cure the devastating disease.	PA2	P11; P17

Most participants would use most of these materials for teaching purposes (71.9%, n = 46). 62.5% (n = 40) of the observation forms indicate that the message perceived during the festival was positive with median overall evaluation of the shorts with a value of 4 (range 1–5).

The message perceived by the participants after the viewing of the shorts and the observation of the debates among the attendees are described by four categories: 1) creative and positive education is possible, 2) awareness of pre-concept and political duties, 3) meaning of life changes the illness experience, 4) family and caregivers also experience suffering.

The categories are substantiated using ideas projected in the short films and registered by the participants. The data is codified by short film theme code (see Table 1).

### 4.1 Creative and positive education is possible

The majority of messages were transmitted through positive or creative symbolism (symbolic language), using humour, music and dance. In a general way, the sessions transmitted life messages with the clear intention to impact the viewer emotionally. Some participants wrote: “Delirious short film with intense music”, “Again, it shows art and music” (PS6). Humour was seen by the participants as the way to cope with situations and promote the welfare of those who receive that information. The participants state this very clearly: “This film shows the topic using humour.” (M2), “We can see that the patient and the caregiver organize medications (presence of laughter, humour, wellbeing).” (H1), “This scene shows tenderness, love, complicity, melancholy, experiences ... around the table.” (H1).

Suffering was represented in many short films through art, music, painting or dance. The intention was to approach it using art so that the viewer might experience it (feel it) in a personal way. Several short films tried to awaken limited emotions (for example, one short used the image of a bomb that was about to explode, plus accelerated images) to show how the mind of a person with mental illness works. Images and sounds can be tools to produce a particular mood that allows for understanding of the story you wish to tell. As the participants described: “He is an artist, life is slow and silent. Contrast with his personality, he (the main character) has mood swings and hallucinations.” (PS2), “The main character paints with precision.” (PA1).

Many short films used storytelling (personal stories) to educate through experiences. “I think they put a beautiful, real face onto the disease, without tragedy or easy humour” (HI3), expressed one participant while watching a testimony. Personal stories were an authentic way of transmitting information and the participants try to guess the information behind the diffused images: “This patient testimonies on how they (hill people) feel in a waiting room. There are empty chairs. It transmits a message to keep going and do not give up.” (CA3).

### 4.2 Awareness of preconception and political duties

This category includes specific messages, objectified in criticising particular acts and behaviour, contextualized within a time and a culture. The participants described pre-concepts relating to HIV/AIDS short movies - “HIV is frowned upon and other diseases are better looked upon or cause compassion.” (HI3) - denouncing that the homosexuality was still not accepted in some communities, particularly due to religious or cultural issues: “It deals with the issue of AIDS (in Africa) as people die with the disease” (HI5).

Professional training was recognized as the way to improve support: “There is a lack of training in empathy. Healthcare professionals are trained in medical but not in social skills. There is a need for comprehensive training in health services” (PS4). The idea that healthcare professionals could be sensitized and trained to deal with the stigma of disease is registered by the participants that also state that there are an increase in associations to support patients and caregivers: “The idea of having a patients' association (e.g. Alzheimer Friends Association) is very valuable” (PA2).

The shorts portrayed other problems of society, such as the lack of community information to deal with extreme situations. For example, society tends to “relate lack of memory to ageing and not to a mental health problem” (M3).

Especially in the Caregiving and Health Context sessions, the message captured by participants was that there was a problem of polypharmacy. Currently, medication was the “first line of treatment for all people's signs and symptoms” (H1) and, “society, including healthcare professionals, were not prepared to intervene differently” (H1).

HIV/AIDS short films treated this topic critically by recalling the political duties regarding the information and prevention of this disease. One participant wrote while watching the movie: “The patient is distressed by what others may think. There are no public prevention campaigns” (HI4).

Moreover, the Memory session conveyed the message that governments should be more responsible for promoting health, educating society and creating social policies to support caregivers: “The lack of control that one has over his own life is horrible (reflection on social protection)” (M1), “If the primary caregiver fails, who takes care of the patient?” (M3).

### 4.3 The meaning of life changes the illness experience

In this category, the message was related to the inner experience of the person, particularly those portraying suffering and death related topics*:”* Even with this horrible disease, the strength and fragility of this account makes the viewer empathize with the subject. It is real" (PA1). The “total pain” suffered by patients and families was authentically identified in all sessions, particularly in the Parkinson session: “This short is presented as something simple to understand because it is based on a real situation (PA1).

Messages on mental illness were registered in the Psyche session, stating from the outset, that physical and psychological pain, both for the patient and the family/caregiver, were most of time, invisible, unstable, confusing and exasperating. Also, the Caregiving and Memory sessions triggered adverse reactions and feelings in the participants: “Very strong film dominated by the sound of groans” (C1). If on the one hand, the observer visualized suffering, on the other, it was linked to a positive feeling. One participant wrote “Empathy – is the love of others until the end” (C3).

This message was reinforced by the description of symbolic language, expressions, and striking images without sound (e.g. battered naked bodies, muted screams, intense facial expressions and mysterious music), trying to instil in the viewer the real idea that suffering exists and how it feels: “They use of sad music during the short” (M3).

The daily experience of living with disease promoted an internal force that allowed recognition that pain was not inevitable, but treatable. The legacy was perceived as a source of the sense of living. Some shorts showed images of patients with severe disabilities who continued to carry out daily activities supported by family and friends, such as “walking”, “eating ice cream”, “playing sports” and “enjoying nature” (PA1).

Daily experiences were seen as essential attitudes to give meaning to life. “A house with love and life everywhere” (M2), metaphorically, the participants described one of the short films that showed an image of a house filled with objects, collections, portraits and paintings transcribing the importance of focussing on suffering through, objects, memories, love and spirituality.

The shorts and audience discussions afterwards, particularly in the Cancer and Context sessions, prompted a particular view that there was place for life in the course of disease. Time is precious and there is much more to do than waiting for death. One participant wrote “It is essential to take advantage of the time we have, taking the opportunity to know other things” (CA1).

Several participants also commented on a current social paradigm, mainly represented in the cancer session related to values: “The short promotes reflections on values in the face of illness” (CA3). In the films, cancer patients were not allowed to talk about some topics with family or even healthcare professionals - “Patients” speeches are interrupted - then they are clarified and the relations between them (patient and family) become entangled again" (CA3) - even when they expressed the need to think about legacy, family burden or fear of death.

### 4.4 Family and caregivers also experience suffering

This category includes the messages perceived on the role (physical/practical, psychological/emotional, social and spiritual) of family and caregivers. This group of people was identified as the pillar of patient well-being: “There is nothing more powerful than love. (The patient) has a wife and he will have a son” (PS1), stated a participant. The illness inflicted suffering on the caregivers, who in most cases ended up exhausted. Even recognizing that caregivers lost several personal and social roles, they were still seen as the pillar of the person with an illness. The idea that the consequences of the disease affected family members stood out in all the sessions: “Families and caregivers stay with their patients even when they are fighting against burnout” (C4).

Another social paradigm was underlined. Caregivers are the social group that keeps the patient well looked after; however, they are also being transformed into patients: “The carer (old man) paints his wife's nails”; “the carer falls in the bathroom and needs help. He is alone” (M1). Several scenes (particularly during the Memory session) portrayed the caregiver as the person who took care of all the primary and emotional care of the patient, and who took care of him/herself with no family and social resources: “The patient sleeps on the sofa (...) a true-to-life portrait of a caregiver (...) exhaustion (...) loss of identity (...) works from home (...) his mother falls and moans while she tries to work” (C2).

In most films the harmony, tenderness, love and cooperation between the patient and the caregiver was identified by the participants as a facilitator to endure the disease context: “Caregiving only makes sense if you do it with love” (C2).

## 5 Discussion

The perceived messages about different illnesses, such as HIV AIDS, dementia (Alzheimer's) and cancer in short films, were categorized into four categories: 1) creative and positive education is possible, 2) awareness of preconception and political duties, 3) meaning of life changes the illness experience, 4) family and caregivers also experience suffering.

The participants understood that the short films encouraged the general public to analyse stigmas and the consequences of severe illness, in a short space of time. Personal stories (storytelling) highlighted unfavourable aspects of the disease, and it was understood as a positive way for the communication of stressful situations. This strategy of knowledge transfer, based on intelligent and sensitive advertising, makes it possible to learn positively about complex issues, reducing stigma in society (Rieger et al., 2018).

Despite variations in participants’ professional status and age, the perception of the films coincided in an emotional or positive state. There was a slogan recorded several times in the notes of the participants: “We won by love”. The transmission of emotions and positive messages reached the participants in the same way regardless of their education. For example, the short films increased the understanding that one person's disease has an impact on all the other family members. This impact on family members has earlier been described as “living on hold” (Sandgren et al., 2010) and as adjusted family interaction with the purpose to maximize the time left together (Möllerberg et al., 2017).

The short films enhanced feelings of the understanding and acceptance of the illnesses, as well as the understanding of suffering. The participants identified the pain that the disease caused patients and family and, at the same time, they recognized that suffering (although not avoidable) can be treatable, can be tolerable and can allow us to live with some quality. This effect of audio-visual materials on feelings has been described earlier (Dickens et al., 2018; Martínez et al., 2017). It has also been known for some time that experience gained through visual resources, emotions and listening can be converted into knowledge (Pickersgill et al., 2018; Tjora, 2015). Although Dickens et al. (2018) claim that even if the films promote emotional engagement, the actual learning can be limited. However, it has also been argued that human values can be challenged through images, through the environment, social debate and through multidisciplinary dialogue in an informal event (Carta, 2015; Centeno et al., 2017).

There are few film festivals dedicated to the context of severe disease, open to discussion in society, seeking to inform and educate the population through art. Since the FICAE festival combined all these elements, the audience had the possibility to interact and talk about difficult issues in an open environment. The participants of this study perceived that this combination of viewing films and participating in discussions afterwards may promote a positive message about disease and its context.

Although talking about positive feelings and happiness during an incapacitating illness might be regarded as socially unacceptable and contradictory by some people (Westerlund et al., 2018), the FICAE festival gave the audience the opportunity to talk about these complex issues freely.

As literature indicates, cultural or thematic festivals are events that strengthen interpersonal relationships within a community. Stimuli such as brightness, colour, image and sound also facilitate an environment of well-being (Wilson et al., 2017). In this study, we found that fiction, audio-visual messages and the festival event, are increasingly influential in society and are potent ways to transmit complex messages (Hernandez et al., 2016; Wilson et al., 2017). We argue that this can be a valid strategy for palliative care public awareness empowering the true knowledge of its concept and clarifying stigmas. The lack of initiatives like FICAE, or the lack of opportunity to attend them, can be seen as barriers to the debate and to the transfer of knowledge in the health field, particularly end-of-life topics, such as death and dying, suffering and acceptance without taboos.

Healthcare awareness through audio-visual materials can promote critical thinking within society and for the professionals involved in decision making (Cruz-Oliver et al., 2017; List, 2018). The use of humanities and art has been highlighted as a way to encourage personal growth and as an effective learning tool for health science students (Johnson and Jackson, 2005). However, a literature review from 2015 (Diaz Membrives et al., 2016) concludes that even if cinema is a commonly used method, its usefulness and validity is still lacking. In addition, Drukarczyk et al. (2014) argue that to be a major public educational resource, it is of importance that the movies present a closer presentation of reality. In order to send positive and truthful messages about severe and disabling illness, further research is needed to investigate how and which ways are beneficial to reach the general public.

This study has several limitations that may influence the results. It has a convenience sample from different professionals and settings. Hence, all participants were linked to the clinical area and were predominantly high-educated. It is true that participants are not experts in audio-visual language or fiction, and that is why we chose an observation form for all, although it does not include all the nuances that audio-visual language can show. It would have been beneficial to delve deeper into the concepts which emerged in the questionnaire and to universalize them with the participants before using the instrument. It should be noted that the shorts viewed belonged mainly to European or Western countries. However, the distinct origins have not translated into differences related to human values.

## 6 Conclusions

This study shows that short films in an innovative public event (Festival) had an impact on people's attitudes and on their awareness of illnesses. This event allowed for the promotion of debate on difficult topics and for sensitizing and demystifying society on issues that cause discomfort. It also facilitated pedagogical, scientific awareness and education about the setting of several illnesses. In this regard, universities should promote more these types of events. Students can learn better by empowering their emotions and critical thinking and so, it is essential to delve into innovative learning styles and creative methods, as social events, involving society in the debate. We concluded that audio-visual materials are a powerful tool, using various types of language as image and sound, to communicate an effective and positive message about health issues. It seems to be a valid strategy for knowledge transfers on difficult topics, particularly those related to end-of-life.

## Declarations

### Author contribution statement

Carla Reigada; Salvador Martín-Utrilla, Pilar Pérez-Ros, Carlos Centeno, Anna Sandgren, Beatriz Gómez-Baceiredo: Conceived and designed the experiments; Performed the experiments; Analyzed and interpreted the data; Contributed reagents, materials, analysis tools or data; Wrote the paper.

### Funding statement

This work was supported by the Institute for Culture and Society (ICS) of the University of Navarra, Spain.

### Competing interest statement

The authors declare no conflict of interest.

### Additional information

No additional information is available for this paper.
